# Supplementary material for: The interactome of intact mitochondria by cross-linking mass spectrometry provides evidence for coexisting respiratory supercomplexes
Source: Mol Cell Proteomics. 2017 Dec 8;17(2):216–32. doi: 10.1074/mcp.RA117.000470 (PMC5795388; doi:10.1074/mcp.RA117.000470)
Supplement: Supplemental Data [file supp_RA117.000470_133922_0_supp_23978_fzffwf.zip › spectra_annotation/mito_spectra_annotation/498-1-4-1-10-1.pdf]

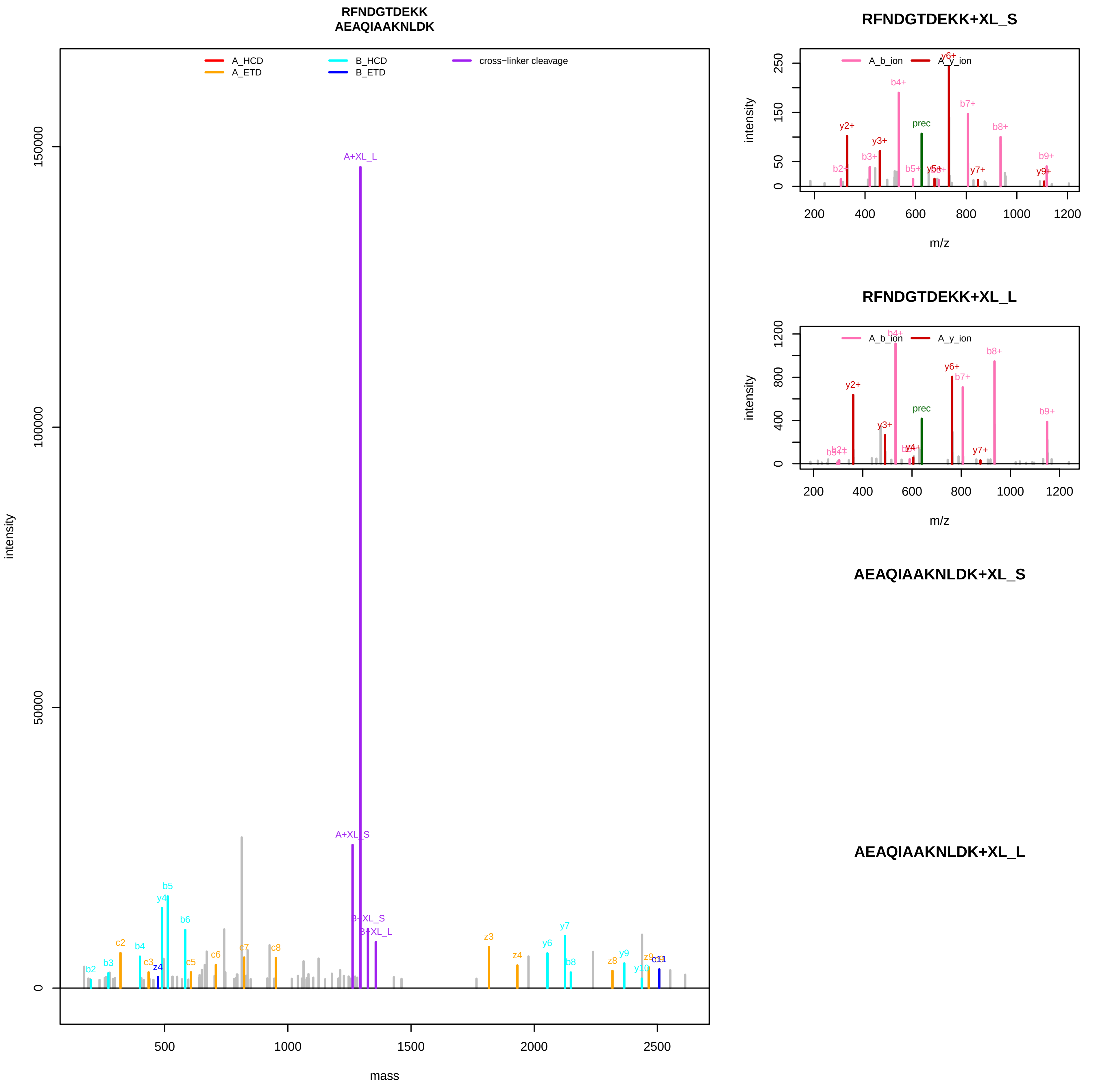
RFNDGTDEKK
AEAQIAAKNLDK
A_HCD
A_ETD
B_HCD
B_ETD
cross−linker cleavage
A+XL_L
A+XL_S
B+XL_S
B+XL_L
intensity
mass
0
50000
100000
150000
500
1000
1500
2000
2500
b2
b3
c2
b4
c3
z4
y4
b5
b6
c5
c6
c7
c8
z3
z4
y6
y7
b8
z8
y9
y10
c11
RFNDGTDEKK+XL_S
A_b_ion
A_y_ion
y6+
b4+
b7+
y2+
prec
b8+
y3+
b3+
b2+
b5+
y5+
y7+
b9+
y9+
m/z
RFNDGTDEKK+XL_L
b4+
b8+
y6+
b7+
y2+
prec
b9+
y3+
y4+
y7+
m/z
AEAQIAAKNLDK+XL_S
AEAQIAAKNLDK+XL_L
